# Impact of tumor size on overall survival and cancer-specific survival of early-onset colon and rectal cancer: a retrospective cohort study

**DOI:** 10.1007/s00384-024-04644-5

**Published:** 2024-05-08

**Authors:** Wanbin Yin, Maorun Zhang, Zhe Ji, Xiaoping Li, Shiyao Zhang, Gang Liu

**Affiliations:** 1 Department of General Surgery, Tianjin Medical University General Hospital, Tianjin, China; 2 Department of Anorectal Surgery, Affiliated Hospital of Jining Medical University, Jining, China

**Keywords:** Colorectal cancer, Cancer-specific survival, Early-onset, Overall survival, Tumor size

## Abstract

**Purpose:**

This study aimed to investigate the impact of tumor size on survival in early-onset colon and rectal cancer.

**Methods:**

Early-onset colon and rectal cancer patients were identified from the Surveillance, Epidemiology, and End Results (SEER) database between 2004 and 2015. Tumor size was analyzed as both continuous and categorical variables. Several statistical techniques, including restricted cubic spline (RCS), Cox proportional hazard model, subgroup analysis, propensity score matching (PSM), and Kaplan–Meier survival analysis, were employed to demonstrate the association between tumor size and overall survival (OS) and cancer-specific survival (CSS) of early-onset colon and rectal cancer.

**Results:**

Seventeen thousand five hundred fifty-one (76.7%) early-onset colon and 5323 (23.3%) rectal cancer patients were included. RCS analysis confirmed a linear association between tumor size and survival. Patients with a tumor size > 5 cm had worse OS and CSS, compared to those with a tumor size ≤ 5 cm for both early-onset colon and rectal cancer. Notably, subgroup analysis showed that a smaller tumor size (≤ 50 mm) was associated with worse survival in stage II early-onset colon cancer, although not statistically significant. After PSM, Kaplan–Meier survival curves showed that the survival of patients with tumor size ≤ 50 mm was better than that of patients with tumor size > 50 mm.

**Conclusion:**

Patients with tumors larger than 5 cm were associated with worse survival in early-onset colon and rectal cancer. However, smaller tumor size may indicate a more biologically aggressive phenotype, correlating with poorer survival in stage II early-onset colon cancer.

**Supplementary Information:**

The online version contains supplementary material available at 10.1007/s00384-024-04644-5.

## Introduction

Colorectal cancer (CRC) is a major health concern that is the second most deadly and third most common cancer worldwide [1]. Early-onset CRC, defined as CRC diagnosed in individuals < 50 years of age, has been on a concerning rise globally in the past decades [2–9]. Clinical features of early-onset CRC differ from those of later-onset disease [4, 10]. A deeper understanding of characteristics in early-onset CRC is highly warranted.

Recent studies have begun to shed light on the unique genetic, clinicopathological, and molecular characteristics of early-onset CRC compared to its later-onset counterpart. These studies have unveiled differences in genetic mutations [11–14], lifestyle factors [15–17], gut microbiome [18]. Despite this growing body of research, there remains a significant gap in our understanding of how tumor size affects survival outcomes in young patients with CRC. Although tumor size has been recognized as a prognostic factor for CRC, the results were inconsistent [19–23]. To the best of our knowledge, no study has investigated the impact of tumor size on survival outcomes in early-onset CRC.

This study aimed to bridge this knowledge gap by examining the impact of tumor size on the survival of patients with early-onset CRC. We hypothesize that tumor size may have a distinctive role in the prognosis of these patients, potentially influencing treatment decisions and survival outcomes differently than in older populations. Through a comprehensive analysis of clinical data from the Surveillance, Epidemiology, and End Results (SEER) database, this study seeks to provide new insights into the prognostic significance of tumor size in early-onset CRC, thereby contributing to more tailored and effective treatment strategies and improving survival outcomes and quality of life for this unique patient demographic.

## Materials and methods

### Study design and participants

This was a retrospective cohort study. We used SEER*Stat 8.4.1 software and selected “Incidence - SEER Research Plus Data, 18 Registries, Nov 2020 Sub (2000–2018)” as the database. The clinicopathological data of patients diagnosed with early-onset CRC between 2004 and 2015 were extracted from the abovementioned database. Primary tumor sites (C18.0, C18.2–18.7, C19.9, and C20.9) included the colon (cecum, ascending colon, hepatic flexure of colon, and transverse colon, splenic flexure of colon, descending colon, sigmoid colon, and rectosigmoid junction), and rectum. In addition, the histologic subtypes included adenocarcinoma (8140/3, 8144/3, 8201/3, 8210/3, 8211/3, 8213/3, 8220/3, 8221/3, 8255/3, 8260/3, 8261/3, 8262/3, 8263/3, 8310/3, 8323/3), mucinous adenocarcinoma (MA) (8480/3, 8481/3), and signet ring cell carcinoma (SRCC) (8490/3). Cases were coded according to the International Classification of Diseases for Oncology, Third Edition.

The inclusion criteria were patients diagnosed with early-onset CRC (pathologically confirmed) between 2004 and 2015. Exclusion criteria were as follows: patients whose tumor size was 0, unknown, or larger than 200 mm; patients who did not undergo surgery; patients with a loss of vital clinical and survival information; and patients younger than 18 years old.

This study followed the Strengthening the Reporting of Cohort Studies in Surgery (STROCSS) reporting guidelines [24].

### Study variables

The collected variables included age, sex, race, tumor size, histologic subtypes, grade, stage, chemotherapy, survival time, cause of death, and vital status records. The endpoint of this study was overall survival (OS) and cancer-specific survival (CSS). In the SEER database, patients between 2004 and 2010 were classified with the sixth American Joint Committee on Cancer (AJCC) classification, and patients between 2010 and 2015 were classified with both the sixth and seventh classification. Thus, to unify the criteria, all patients were classified according to the sixth AJCC classification. OS was defined as the time from diagnosis to death from any cause or the last follow-up. CSS was defined as the time interval between cancer diagnosis and death from colorectal cancer or the last follow-up.

### Statistical analysis

Colon cancer is studied separately from rectal cancer. X-tile software [25] (version 3.6.1, Yale University School of Medicine) was used to determine the optimal cut-off points for age. Categorical variables were expressed as frequencies and percentages. OS and CSS were analyzed using Kaplan–Meier curves and compared using log-rank tests.

Potential nonlinear associations between tumor size and outcomes were examined using restricted cubic spline (RCS) [26] with 4 knots. Covariates included in the analysis were age, sex, race, histologic subtypes, grade, stage, and chemotherapy.

Univariate and multivariate Cox regression analyses were performed to calculate hazard ratios (HR) and 95% confidence intervals (CI). To fully assess the relationship between tumor size and outcomes, tumor size was analyzed as both continuous and categorical variables (two, three, and four categories). Cut-off values for tumor size were determined based on professional experience and X-tile software.

Additionally, based on 3 models, the *P* values for linear trends were calculated using the quartile values as an ordinal variable. Model 1 was unadjusted; Model 2 was adjusted for age, sex, and race; Model 3 was further adjusted for histologic subtypes, grade, stage, and chemotherapy.

To assess the consistency of the impact of tumor size on outcomes, subgroup analysis was performed according to the above-mentioned covariates. Moreover, likelihood ratio tests were used to examine interaction [27].

To reduce the impact of baseline differences on the outcomes, a sensitivity analysis was carried out using 1:1 propensity score matching (PSM) [28]. The balance in covariates was assessed by using the standardized mean difference (SMD) approach. SMD of 10% or less was considered to be adequate balance. After PSM, OS and CSS were analyzed using Kaplan–Meier curves and log-rank tests.

R software (version 4.3.1; http://www.r-project.org) was used for statistical analyses. Two-sided *P* < .05 indicated statistical significance.

## Results

### Characteristics of the participants

**Fig. 1:**
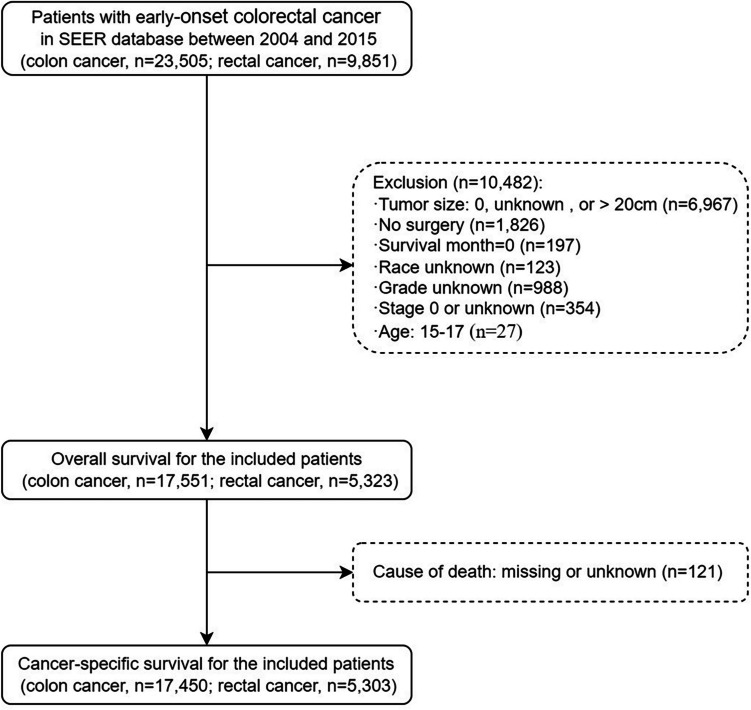
Flow chart of the study population

Overall, 33,356 patients with early-onset colon and rectal cancer were identified between 2004 and 2015 from the SEER database. According to the inclusion and exclusion criteria, 17,551 (76.7%) colon and 5323 (23.3%) rectal cancer patients were included. The study screening flow chart is shown in Fig. 1. The demographic and clinicopathological characteristics of early-onset colon and rectal cancer patients are summarized in Table 1.

**Table 1:** Demographic and clinicopathological characteristics of patients with early-onset colon and rectal cancer

**Variables**	**Colorectal cancer**	**Colon cancer**	**Rectal cancer**	***P***
	**(*n*** **= 22874)**	**(*n*** **= 17551)**	**(*n*** **= 5323)**	
Age^a^ (years)	44 (39–47)	44 (39–47)	44 (39–47)	0.606
Sex				< 0.001
Female	10820 (47.3)	8546 (48.7)	2274 (42.7)	
Male	12054 (52.7)	9005 (51.3)	3049 (57.3)	
Race				< 0.001
Black	3275 (14.3)	2778 (15.8)	497 (9.3)	
Other	2593 (11.3)	1959 (11.2)	634 (11.9)	
White	17006 (74.3)	12814 (73.0)	4192 (78.8)	< 0.001
Tumor size^a^ (mm)	45.5 (33–60)	50 (35–65)	40 (26–55)	< 0.001
Histologic types				< 0.001
Adenocarcinoma	20549 (89.8)	15636 (89.1)	4913 (92.3)	
MA/SRCC	2325 (10.2)	1915 (10.9)	410 (7.7)	
Grade				< 0.001
I	1527 ( 6.7)	1155 (6.6)	372 (7.0)	
II	16544 (72.3)	12507 (71.3)	4037 (75.8)	
III	4165 (18.2)	3347 (19.1)	818 (15.4)	
IV	638 ( 2.8)	542 (3.1)	96 (1.8)	
Stage				< 0.001
I	3315 (14.5)	2215 (12.6)	1100 (20.7)	
II	5742 (25.1)	4640 (26.4)	1102 (20.7)	
III	9156 (40.0)	6715 (38.3)	2441 (45.9)	
IV	4661 (20.4)	3981 (22.7)	680 (12.8)	
Chemotherapy				< 0.001
No/Unknown	7489 (32.7)	6458 (36.8)	1031 (19.4)	
Yes	15385 (67.3)	11093 (63.2)	4292 (80.6)	< 0.001
Survival months^a^	67 (38–114)	66 (36–113)	72 (44–117)	
Survival status				< 0.001
Alive	15096 (66.0)	11374 (64.8%)	3722 (69.9%)	
Dead	7778 (34.0)	6177 (35.2%)	1601 (30.1%)	

*MA* mucinous adenocarcinoma, *SRCC* signet ring cell carcinoma

^a^median (interquartile range)

### Kaplan–Meier survival analysis

**Fig. 2:**
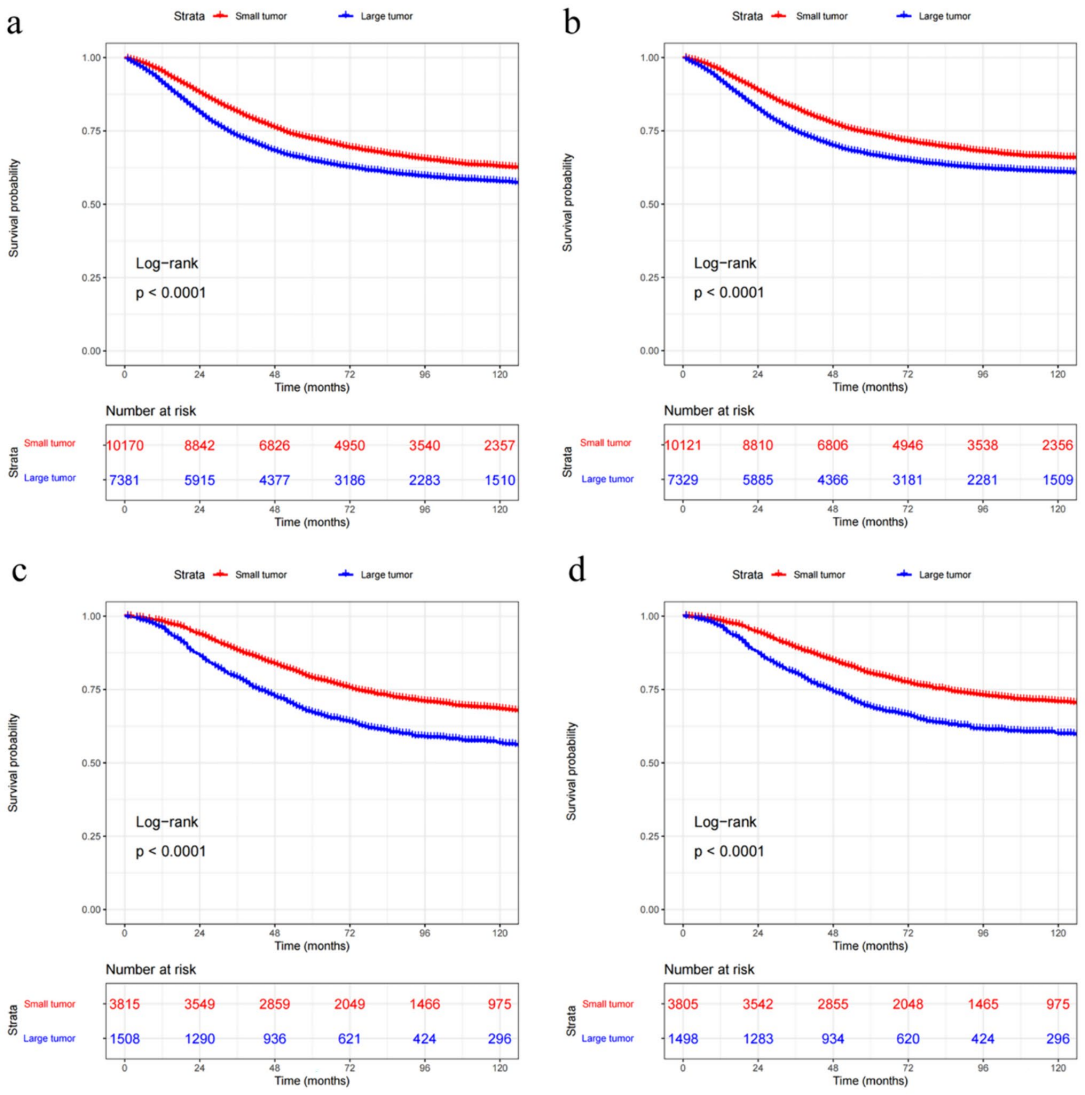
Kaplan–Meier curves for overall survival (**a**) and cancer-specific survival (**b**) of patients with early-onset colon cancer; Kaplan–Meier curves for overall survival (**c**) and cancer-specific survival (**d**) of patients with early-onset rectal cancer. Large tumor: >50 mm; Small tumor: ≤ 50 mm

The 3-, 5‐, and 10‐year OS rates were 78.0%, 69.1%, and 60.7%, respectively, and the 3‐, 5‐, and 10‐year CSS rates were 79.3%, 71.0%, and 63.9%, respectively for early-onset colon cancer. The 3‐, 5‐, and 10‐year OS rates were 85.7%, 75.7%, and 65.2%, respectively, and the 3‐, 5‐, and 10‐year CSS rates were 86.8%, 77.3%, and 67.9%, respectively for early-onset rectal cancer. The OS and CSS in patients with tumor size ≤ 50 mm were better compared with those with tumor size > 50 mm (Fig. 2).

### Potential nonlinear associations between tumor size and survival

**Fig. 3:**
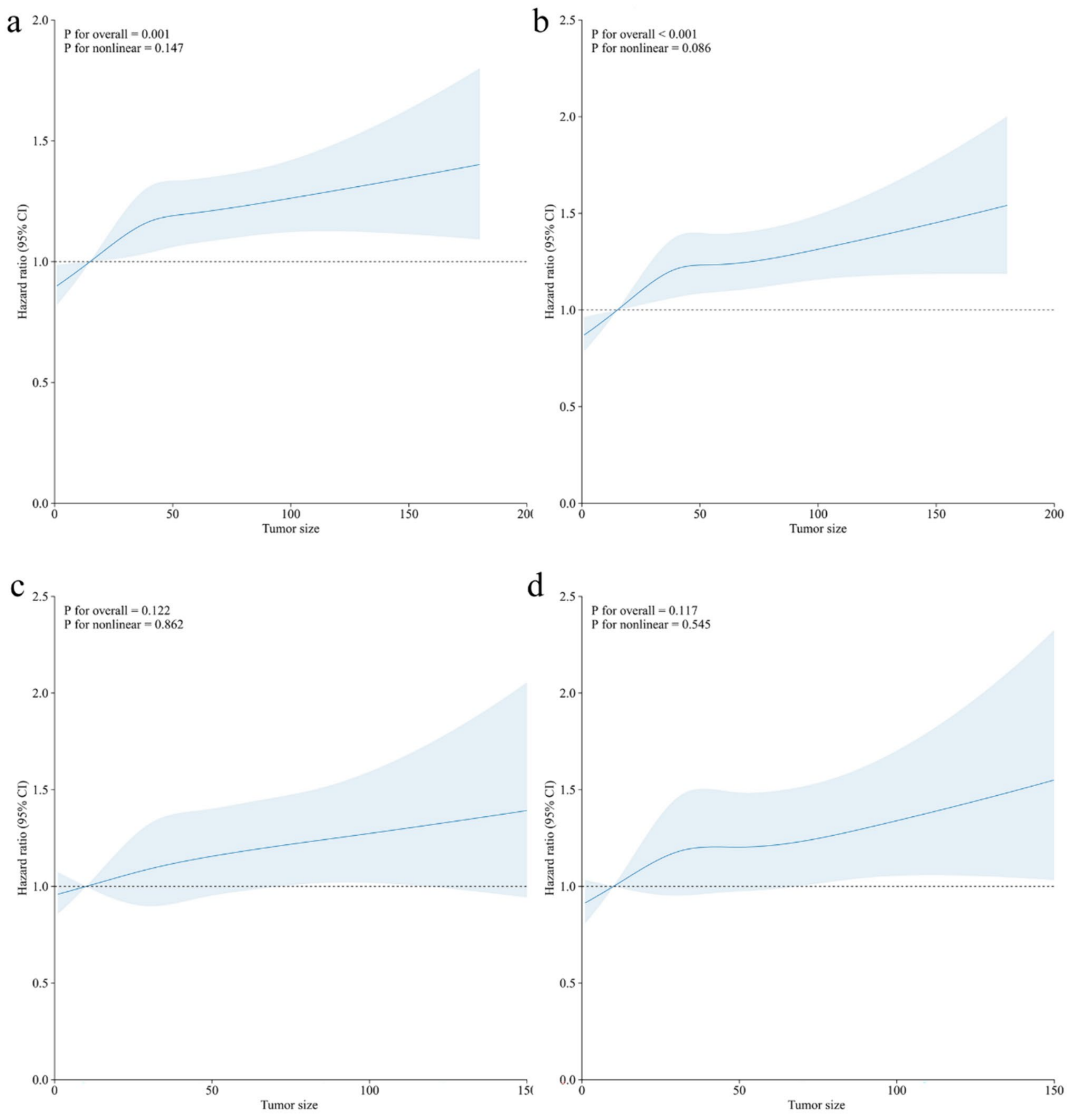
Association between tumor size and survival using a restricted cubic spline regression model. Early-onset colon cancer: **a** overall survival; **b** cancer-specific survival. Early-onset rectal cancer: **c** overall survival; **d** cancer-specific survival. Graphs show HRs for survival according to tumor size adjusted for age, sex, race, histologic types, grade, stage, and chemotherapy. Data were fitted by a restricted cubic spline Cox proportional hazards regression model, and the model was conducted with 4 knots at the 5th, 35th, 65th, 95th percentiles of tumor size (reference is the 5th percentile). Solid lines indicate HRs, and shadow shape indicate 95% CIs. HR, hazard ratio; CI, confidence interval

The RCS revealed that the risk of OS and CSS increased linearly with increasing tumor size. (Fig. 3).

### Prognostic impact of tumor size on OS and CSS

Tumor size had a negative impact on OS and CSS in both the unadjusted model and the fully adjusted model, regardless of whether it was analyzed as a continuous or categorical variable (Table 2).

**Table 2:** Association between tumor size and survival in early-onset colon and rectal cancer

	**Adjusted HR**^a^ **(95% CI)**			
**Tumor size (mm)**	**Colon cancer**		**Rectal cancer**	
	**OS**	**CSS**	**OS**	**CSS**
Continuous variable	1.002 (1.001–1.003)	1.002 (1.001–1.003)	1.003 (1.001–1.005)	1.003 (1.001–1.005)
Categorical variable				
≤ 20	Reference	Reference	Reference	Reference
> 20	1.212 (1.069–1.374)	1.268 (1.104–1.457)	1.113 (0.950–1.305)	1.146 (0.965–1.361)
≤ 30	Reference	Reference	Reference	Reference
> 30	1.097 (1.020–1.179)	1.114 (1.032–1.203)	1.069 (0.954–1.197)	1.067 (0.946–1.203)
≤ 40	Reference	Reference	Reference	Reference
> 40	1.072 (1.014–1.132)	1.076 (1.016–1.140)	1.125 (1.016–1.247)	1.100 (0.988–1.225)
≤ 50	Reference	Reference	Reference	Reference
> 50	1.070 (1.016–1.126)	1.064 (1.008–1.124)	1.169 (1.052–1.298)	1.138 (1.020–1.271)
≤ 24^b^	Reference	Reference	Reference	Reference
24–55	1.187 (1.058–1.332)	1.233 (1.087–1.398)	1.067 (0.919–1.240)	1.095 (0.933–1.286)
≥ 55	1.280 (1.138–1.440)	1.316 (1.158–1.497)	1.215 (1.033–1.430)	1.213 (1.020–1.442)
≤ 20	Reference	Reference	Reference	Reference
> 20, ≤ 4 0	1.176 (1.032–1.340)	1.229 (1.064–1.420)	1.052 (0.887–1.247)	1.101 (0.916–1.322)
> 40, ≤ 60	1.218 (1.070–1.385)	1.275 (1.105–1.470)	1.148 (0.964–1.366)	1.166 (0.967–1.406)
> 60	1.259 (1.103–1.438)	1.314 (1.136–1.520)	1.206 (1.001–1.454)	1.218 (0.997–1.487)

*HR* hazard ratios, *CI* confidence interval, *OS* overall survival, *CSS* cancer-specific survival

^a^adjusted for age, sex, race, histologic types, grade, stage, and chemotherapy

^b^The cut-off values were determined by the X-tile software

### Linear trend analysis

When tumor size was categorized based on quartiles, it still negatively impacted OS and CSS. Additionally, *P*-values for linear trend were significant in all 3 models for early-onset colon cancer, and significant in Model 1 and Model 2 for early-onset rectal cancer (Table 3).

**Table 3:** Association between tumor size and survival in early-onset colon and rectal cancer (according to quartile of tumor size)

	**HR (95% CI)**				
	**Q1**	**Q2**	**Q3**	**Q4**	***P*** **for Trend**
**Colon cancer**^a^					
OS					
Model 1	Reference	1.398 (1.305–1.498)	1.500 (1.391–1.617)	1.581 (1.469–1.701)	< 0.001
Model 2	Reference	1.396 (1.303–1.496)	1.488 (1.380–1.605)	1.568 (1.456–1.688)	< 0.001
Model 3	Reference	1.055 (0.984–1.132)	1.092 (1.011–1.179)	1.117 (1.035–1.205)	0.003
CSS					
Model 1	Reference	1.460 (1.357–1.570)	1.544 (1.426–1.672)	1.630 (1.509–1.762)	< 0.001
Model 2	Reference	1.457 (1.354–1.567)	1.533 (1.416–1.661)	1.620 (1.498–1.752)	< 0.001
Model 3	Reference	1.069 (0.993–1.150)	1.093 (1.008–1.185)	1.123 (1.036–1.217)	0.005
**Rectal cancer**^b^					
OS					
Model 1	Reference	1.233 (1.064–1.430)	1.680 (1.448–1.949)	1.946 (1.685–2.247)	< 0.001
Model 2	Reference	1.229 (1.060–1.425)	1.666 (1.436–1.933)	1.890 (1.636–2.185)	< 0.001
Model 3	Reference	0.944 (0.812–1.098)	1.082 (0.927–1.261)	1.090 (0.936–1.269)	0.071
CSS					
Model 1	Reference	1.339 (1.145–1.567)	1.782 (1.521–2.088)	2.071 (1.775–2.415)	< 0.001
Model 2	Reference	1.334 (1.140–1.541)	1.766 (1.507–2.070)	2.001 (1.723–2.348)	< 0.001
Model 3	Reference	0.987 (0.842–1.159)	1.091 (0.926–1.285)	1.092 (0.929–1.283)	0.135

Model 1: unadjusted; Model 2: adjusted for age, sex, and race; Model 3: adjusted for age, sex, race, histologic types, grade, stage, and chemotherapy

*Q* quartile, *HR* hazard ratios, *CI* confidence interval, *OS* overall survival, *CSS* cancer-specific survival

^a^Q1: 1–35, Q2: 36–50, Q3: 51–65, Q4: > 65 (mm)

^b^Q1: 1–26, Q2: 27–40, Q3: 41–55, Q4: > 55 (mm)

### Subgroup analysis

**Fig. 4:**
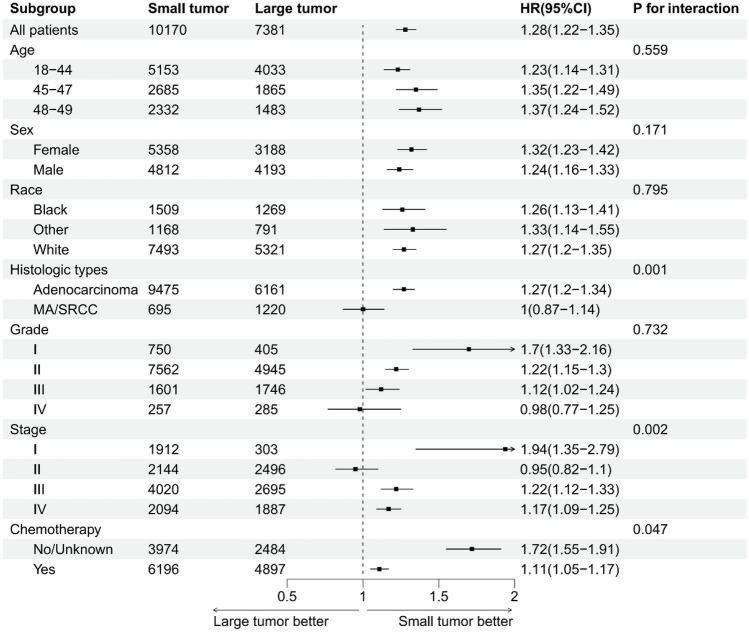
Forest plot for subgroup analysis of overall survival in early-onset colon cancer. Large tumor: > 50 mm; Small tumor: ≤ 50 mm. HR, hazard ratio; CI, confidence interval; MA, mucinous adenocarcinoma; SRCC, signet ring cell carcinoma

**Fig. 5:**
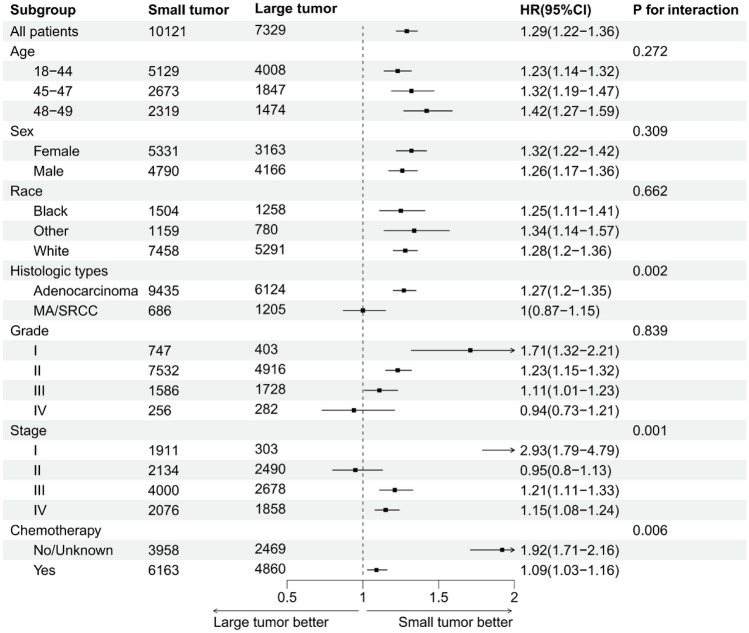
Forest plot for subgroup analysis of cancer-specific survival in early-onset colon cancer. Large tumor: > 50 mm; Small tumor: ≤ 50 mm. HR, hazard ratio; CI, confidence interval; MA, mucinous adenocarcinoma; SRCC, signet ring cell carcinoma

The results of subgroup analysis of OS and CSS in early-onset rectal cancer were consistent (Supplementary Figs. S1 and S2). Particularly notable was the distinct survival advantage observed with larger tumors in Stage II early-onset colon cancer, contrasting with other stages. Although the HRs for Stage I, III, and IV were greater than 1, Stage II presented an HR less than 1 (HR, 0.95; 95% CI, 0.82–1.10), suggesting a unique trend where larger tumor sizes in this stage were associated with better OS compared to smaller tumors (Fig. 4). Similar results were observed in subgroup analysis for CSS (Fig. 5).

### Propensity score matching

**Fig. 6:**
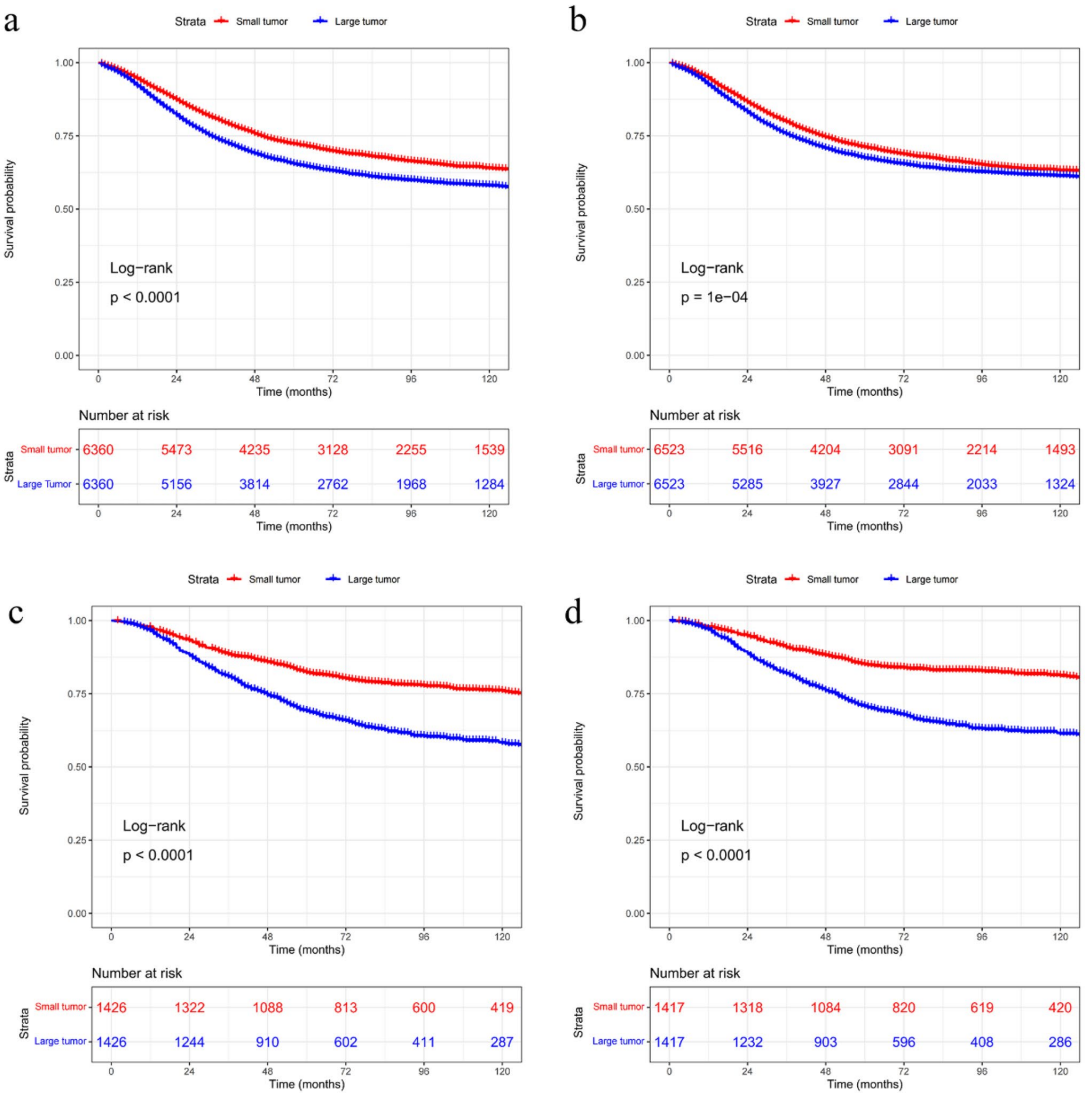
Kaplan–Meier curves for overall survival (**a**) and cancer-specific survival (**b**) of patients with early-onset colon cancer after PSM; Kaplan–Meier curves for overall survival (**c**) and cancer-specific survival (**d**) of patients with early-onset rectal cancer after PSM. Large tumor: > 50 mm; Small tumor: ≤ 50 mm. PSM, propensity score matching

Before PSM, there was a significant imbalance in baseline characteristics. After PSM, no statistically significant differences remained in the covariates (all SMDs < 0.1) for OS and CSS analysis in both early-onset colon (Supplementary Tables S1 and S2) and rectal cancer (Supplementary Tables S3 and S4). After matching, Kaplan–Meier survival curves showed that the prognosis of patients with tumor size ≤ 50 mm was better than that of patients with tumor size > 50 mm (Fig. 6).

## Discussion

Based on the SEER database, 22,874 participants with early-onset colon and rectal cancer were included. Our study revealed that larger tumor size (as both continuous and categorical variables), significantly correlated with worse OS and CSS in early-onset colon and rectal cancer patients. Notably, smaller tumor size was associated with worse survival in stage II early-onset colon cancer (adjusted HR, 0.95; 95% CI, 0.82–1.10, and adjusted HR, 0.95; 95% CI, 0.80–1.13 for OS and CSS, respectively), suggesting that smaller tumors may reflect a more biologically aggressive phenotype for patients with stage II early-onset colon cancer.

In accordance with our findings, some studies have revealed that patients with larger tumors had a decreased survival compared with those with smaller tumors in CRC no matter with [20, 29, 30] or without [31] metastasis. However, there were also studies with different opinions. Hajibandeh et al. evaluated the predictive significance of tumor size in 192 CRC patients undergoing curative surgery [21]. They found that tumor size on its own may not have a significant prognostic value in OS. Their study was limited by the small sample size. In addition, Shiraishi et al. performed a retrospective study of 95 patients with pT4 CRC and demonstrated that tumor size ≥ 50 mm was associated with a better CSS than that of < 50 mm [23]. This contrasting view should be interpreted with caution because only 95 patients were included in the analysis. Overall, we still believe that larger tumor size is associated with worse survival outcomes for CRC.

It should be noted that an interesting result was observed after subgroup analysis. Surprisingly, we identified that tumor size > 50 mm was associated with a better OS and CSS than that of ≤ 50 mm for patients with stage II colon cancer; however, this was not statistically significant. This finding contrasted with other stages and highlighted the nuanced impact of tumor size on survival, depending on the stage of the disease. After a thorough literature search, we found that previous studies have revealed this seemingly paradoxical finding [23, 32–37]. For example, Huang et al. analysed 7719 patients with stage II colon cancer from the SEER database and indicated that patients with smaller tumors were associated with decreased CSS compared with those with larger tumors [32]. This was extremely similar to what we reported here. It was speculated that smaller tumors with heavy intestinal wall invasion may represent a biologically aggressive phenotype, whereas larger tumors may reflect a biologically indolent phenotype in stage II CRC. This distinct growth pattern may be caused by inter-tumor heterogeneity of CRC that results from various genetic and epigenetic factors. More studies are needed to elucidate the underlying mechanism.

There are several strengths to our study. In addition to the large sample size, the main strength of our study was the multiple rigorous statistical methods. First, colon cancer is studied separately from rectal cancer due to their different biological behaviors. Second, potential nonlinear associations between tumor size and outcomes (OS and CSS) were evaluated using RCS. Third, to correct for potential confounding factors, univariable and multivariable Cox regression were used. Additionally, PSM analysis was also performed as a sensitivity analysis. Therefore, the findings of our study were robust. Fourth, tumor size was analyzed as both continuous and categorical variables. Moreover, when it was analysed as a categorical variable, different numbers of categories (two, three, and four categories) and cut-off values were used. Thus, the results were reliable. Fifth, both OS and CSS were evaluated as survival outcomes. Last, subgroup analyses were conducted and a special population was identified (stage II early-onset colon cancer).

However, when interpreting the results of the present study, several limitations should be considered. First, the retrospective nature of the study limited the generalizability of the results. Prospective studies are needed in the future. Second, besides genetics and epigenetics data, microsatellite instability status, comorbidities, intestinal obstruction or penetration, and detailed information on CEA, radiotherapy, and chemotherapy were not included in the SEER database. Third, there is still a possibility of residual confounding, despite adjusting for potential confounders. Fourth, only the US population were included in the SEER database, possibly resulting in a degree of selection bias. The results of the present study might be unsuitable for patients in other countries, suggesting that a large-scale multicenter global study is necessary. Fifth, only patients who had undergone surgical resection were included in this study. Therefore, our results may not apply to patients without surgical resection.

To the best of our knowledge, this is the first large study to reveal the association between tumor size and survival outcomes in early-onset colon and rectal cancer. Our study highlights that tumor size is an important risk factor for OS and CSS in early-onset colon and rectal cancer. More prospective multicenter studies are needed to validate the association between tumor size and survival in stage II early-onset colon cancer, especially stratified by microsatellite instability status. Further studies should also be undertaken to elucidate the underlying genetic and molecular mechanisms of the impact of tumor size on the survival of early-onset colon and rectal cancer.

## Conclusions

This study found that patients with larger tumors experienced worse OS and CSS compared to those with smaller tumors in early-onset colon and rectal cancer. Notably, smaller tumors may reflect a more biologically aggressive phenotype and be associated with worse survival in stage II early-onset colon cancer. More studies are warranted to verify our findings and elucidate the underlying mechanisms.

## Supplementary Information

Below is the link to the electronic supplementary material.

Supplementary file1 (DOCX 760 kb)

## Data Availability

The data sets analyzed in this study are available in the SEER database (https://seer.Cancer.gov/).

## References

[1] Sung H, Ferlay J, Siegel RL et al (2021) Global cancer statistics 2020: GLOBOCAN estimates of incidence and mortality worldwide for 36 cancers in 185 countries. CA Cancer J Clin 71:209–249. 10.3322/caac.21660 33538338 10.3322/caac.21660

[2] Patel SG, Karlitz JJ, Yen T et al (2022) The rising tide of early-onset colorectal cancer: a comprehensive review of epidemiology, clinical features, biology, risk factors, prevention, and early detection. Lancet Gastroenterol Hepatol 7:262–274. 10.1016/S2468-1253(21)00426-X 35090605 10.1016/S2468-1253(21)00426-X

[3] Shah RR, Millien VO, Costa WL et al (2022) Trends in the incidence of early-onset colorectal cancer in all 50 United States from 2001 through 2017. Cancer 128:299–310. 10.1002/cncr.33916 34529823 10.1002/cncr.33916

[4] Sinicrope FA (2022) Increasing incidence of early-onset colorectal cancer. N Engl J Med 386:1547–1558. 10.1056/NEJMra2200869 35443109 10.1056/NEJMra2200869

[5] Ugai T, Sasamoto N, Lee H-Y et al (2022) Is early-onset cancer an emerging global epidemic? Current evidence and future implications. Nat Rev Clin Oncol 19:656–673. 10.1038/s41571-022-00672-8 36068272 10.1038/s41571-022-00672-8 PMC9509459

[6] Ben-Aharon I, van Laarhoven HWM, Fontana E et al (2023) Early-onset cancer in the gastrointestinal tract is on the rise-evidence and implications. Cancer Discov 13:538–551. 10.1158/2159-8290.CD-22-1038 36757194 10.1158/2159-8290.CD-22-1038

[7] Giannakis M, Ng K (2023) A common cancer at an uncommon age. Science 379:1088–1090. 10.1126/science.ade7114 36927016 10.1126/science.ade7114

[8] Siegel RL, Wagle NS, Cercek A et al (2023) Colorectal cancer statistics, 2023. CA Cancer J Clin 73:233–254. 10.3322/caac.21772 36856579 10.3322/caac.21772

[9] Spaander MCW, Zauber AG, Syngal S et al (2023) Young-onset colorectal cancer. Nat Rev Dis Primer 9:21. 10.1038/s41572-023-00432-7 10.1038/s41572-023-00432-7 PMC10589420 37105987

[10] REACCT Collaborative, Zaborowski AM, Abdile A et al (2021) Characteristics of early-onset vs late-onset colorectal cancer: a review. JAMA Surg 156:865–874. 10.1001/jamasurg.2021.2380 34190968 10.1001/jamasurg.2021.2380

[11] Nakamura K, Hernández G, Sharma GG et al (2022) A liquid biopsy signature for the detection of patients with early-onset colorectal cancer. Gastroenterology 163:1242–1251e2. 10.1053/j.gastro.2022.06.089 35850198 10.1053/j.gastro.2022.06.089 PMC9613521

[12] Shen X, DeWan AT, Johnson CH (2023) Early-onset colorectal cancer somatic gene mutations by population subgroups. Cancer Discov 13:530–531. 10.1158/2159-8290.CD-22-1464 36855917 10.1158/2159-8290.CD-22-1464

[13] Seagle HM, Keller SR, Tavtigian SV et al (2023) Clinical multigene panel testing identifies racial and ethnic differences in germline pathogenic variants among patients with early-onset colorectal cancer. J Clin Oncol 41:4279–4289. 10.1200/JCO.22.02378 37319387 10.1200/JCO.22.02378 PMC10852379

[14] Holowatyj AN, Wen W, Gibbs T et al (2023) Racial/ethnic and sex differences in somatic cancer gene mutations among patients with early-onset colorectal cancer. Cancer Discov 13:570–579. 10.1158/2159-8290.CD-22-0764 36520636 10.1158/2159-8290.CD-22-0764 PMC10436779

[15] Wang G, Liu Z (2023) Alcohol intake associated with increased risk of early-onset colorectal cancer. J Clin Oncol 41:5328. 10.1200/JCO.23.01548 37782884 10.1200/JCO.23.01548

[16] Li H, Boakye D, Chen X et al (2022) Associations of body mass index at different ages with early-onset colorectal cancer. Gastroenterology 162:1088–1097e3. 10.1053/j.gastro.2021.12.239 34914944 10.1053/j.gastro.2021.12.239

[17] O’Sullivan DE, Sutherland RL, Town S et al (2022) Risk factors for early-onset colorectal cancer: a systematic review and meta-analysis. Clin Gastroenterol Hepatol 20:1229–1240e5. 10.1016/j.cgh.2021.01.037 33524598 10.1016/j.cgh.2021.01.037

[18] Kong C, Liang L, Liu G et al (2023) Integrated metagenomic and metabolomic analysis reveals distinct gut-microbiome-derived phenotypes in early-onset colorectal cancer. Gut 72:1129–1142. 10.1136/gutjnl-2022-327156 35953094 10.1136/gutjnl-2022-327156

[19] Balta AZ, Özdemir Y, Sücüllü İ et al (2014) Can horizontal diameter of colorectal tumor help predict prognosis? Ulus Cerrahi Derg 30:115–119. 10.5152/UCD.2014.2701 25931910 10.5152/UCD.2014.2701 PMC4379853

[20] Kornprat P, Pollheimer MJ, Lindtner RA et al (2011) Value of tumor size as a prognostic variable in colorectal cancer: a critical reappraisal. Am J Clin Oncol 34:43–49. 10.1097/COC.0b013e3181cae8dd 20101166 10.1097/COC.0b013e3181cae8dd

[21] Hajibandeh S, Barghash M, Khan RMA et al (2022) Predictive significance of tumour size in patients undergoing curative surgery for colorectal cancer: a retrospective cohort study. Cureus. 10.7759/cureus.26656 35949794 10.7759/cureus.26656 PMC9357253

[22] Mejri N, Dridi M, El Benna H et al (2017) Prognostic value of tumor size in stage II and III colorectal cancer in Tunisian population. Colorectal Cancer 6:113–119. 10.2217/crc-2017-0011

[23] Shiraishi T, Ogawa H, Katayama A et al (2022) Association of tumor size in pathological T4 colorectal cancer with desmoplastic reaction and prognosis. Ann Gastroenterol Surg 6:667–678. 10.1002/ags3.12571 36091306 10.1002/ags3.12571 PMC9444861

[24] Mathew G, Agha R, Albrecht J et al (2021) STROCSS 2021: strengthening the reporting of cohort, cross-sectional and case-control studies in surgery. Int J Surg 96:106165. 10.1016/j.ijsu.2021.106165 34774726 10.1016/j.ijsu.2021.106165

[25] Camp RL, Dolled-Filhart M, Rimm DL (2004) X-tile: a new bio-informatics tool for biomarker assessment and outcome-based cut-point optimization. Clin Cancer Res 10:7252–7259. 10.1158/1078-0432.CCR-04-0713 15534099 10.1158/1078-0432.CCR-04-0713

[26] Desquilbet L, Mariotti F (2010) Dose-response analyses using restricted cubic spline functions in public health research. Stat Med 29:1037–1057. 10.1002/sim.3841 20087875 10.1002/sim.3841

[27] Yates T, Summerfield A, Razieh C et al (2022) A population-based cohort study of obesity, ethnicity and COVID-19 mortality in 12.6 million adults in England. Nat Commun 13:624. 10.1038/s41467-022-28248-1 35110546 10.1038/s41467-022-28248-1 PMC8810846

[28] Austin PC (2011) An introduction to propensity score methods for reducing the effects of confounding in observational studies. Multivar Behav Res 46:399–424. 10.1080/00273171.2011.568786 10.1080/00273171.2011.568786 PMC3144483 21818162

[29] Zhang Q, Li B, Zhang S et al (2023) Prognostic impact of tumor size on patients with metastatic colorectal cancer: a large SEER-based retrospective cohort study. Updat Surg 75:1135–1147. 10.1007/s13304-023-01533-4 10.1007/s13304-023-01533-4 PMC10359205 37202599

[30] Yan Q, Zhang K, Guo K et al (2019) Value of tumor size as a prognostic factor in metastatic colorectal cancer patients after chemotherapy: a population-based study. Future Oncol 15:1745–1758. 10.2217/fon-2018-0785 31038364 10.2217/fon-2018-0785

[31] Dai W, Li Y, Meng X et al (2017) Does tumor size have its prognostic role in colorectal cancer? Re-evaluating its value in colorectal adenocarcinoma with different macroscopic growth pattern. Int J Surg 45:105–112. 10.1016/j.ijsu.2017.07.100 28760707 10.1016/j.ijsu.2017.07.100

[32] Huang B, Feng Y, Zhu L et al (2016) Smaller tumor size is associated with poor survival in stage II colon cancer: an analysis of 7,719 patients in the SEER database. Int J Surg 33:157–163. 10.1016/j.ijsu.2016.07.073 27491969 10.1016/j.ijsu.2016.07.073

[33] Wang Y, Zhuo C, Shi D et al (2015) Unfavorable effect of small tumor size on cause-specific survival in stage IIA colon cancer, a SEER-based study. Int J Colorectal Dis 30:131–137. 10.1007/s00384-014-2056-y 25392257 10.1007/s00384-014-2056-y

[34] Li X, An B, Ma J et al (2019) Prognostic value of the tumor size in resectable colorectal cancer with different primary locations: a retrospective study with the propensity score matching. J Cancer 10:313–322. 10.7150/jca.26882 30719125 10.7150/jca.26882 PMC6360316

[35] Muralidhar V, Nipp RD, Ryan DP et al (2016) Association between very small tumor size and increased cancer-specific mortality in node-positive colon cancer. Dis Colon Rectum 59:187–193. 10.1097/DCR.0000000000000532 26855392 10.1097/DCR.0000000000000532

[36] Huang B, Chen C, Ni M et al (2017) The association between small tumor size and poor survival in T4 mucinous adenocarcinoma of colon without distant metastasis. J Buon 22:170–177 28365951

[37] Huang B, Feng Y, Mo S-B et al (2016) Smaller tumor size is associated with poor survival in T4b colon cancer. World J Gastroenterol 22:6726. 10.3748/wjg.v22.i29.6726 27547015 10.3748/wjg.v22.i29.6726 PMC4970476

