# Supplementary material for: Impact of tumor size on overall survival and cancer-specific survival of early-onset colon and rectal cancer: a retrospective cohort study
Source: Int J Colorectal Dis. 2024 May 8;39(1):69. doi: 10.1007/s00384-024-04644-5 (PMC11078790; doi:10.1007/s00384-024-04644-5)
Supplement: Supplementary file 1 — Supplementary file1 (DOCX 760 kb) [file 384_2024_4644_MOESM1_ESM.docx]

Supplementary Material


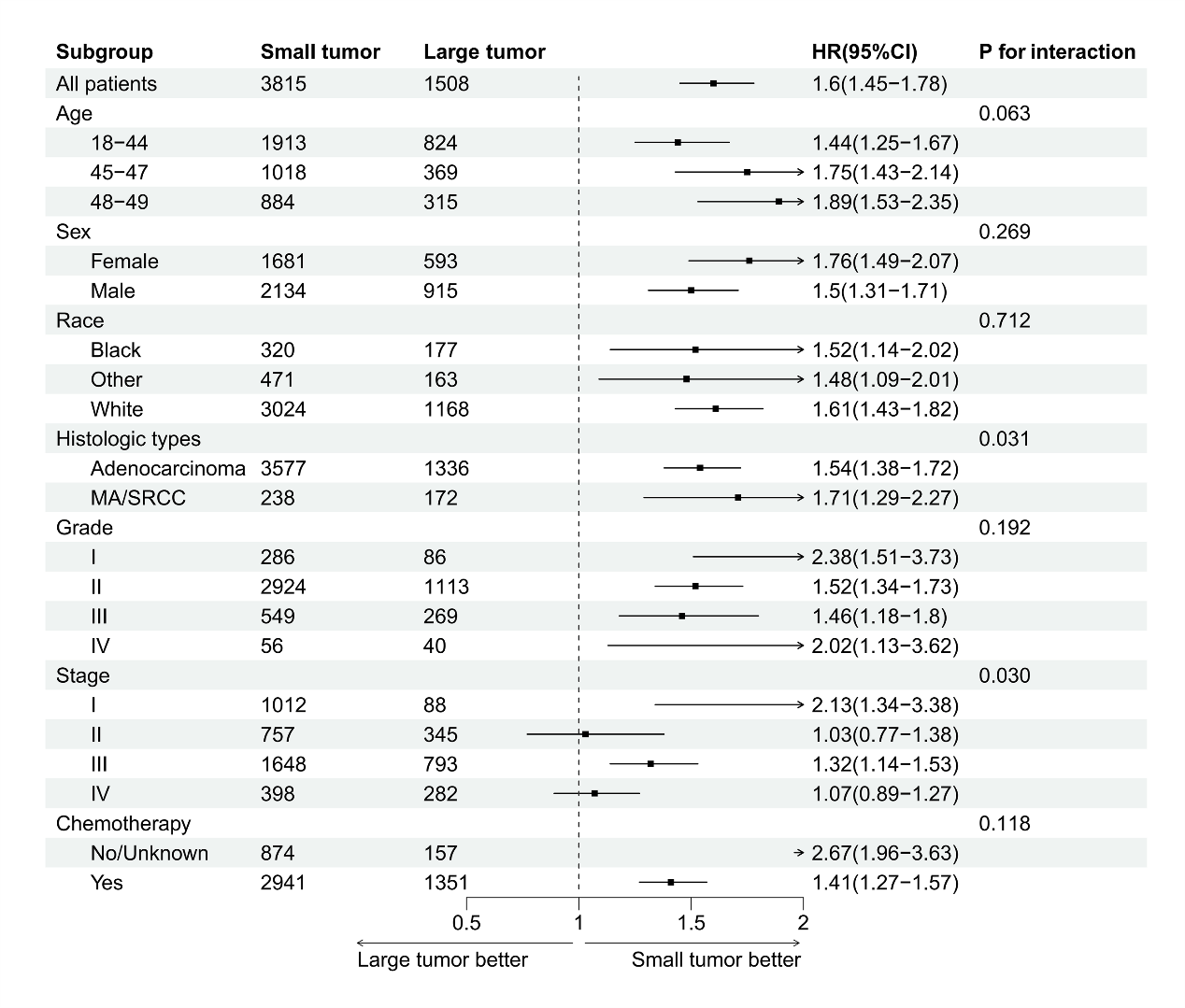


**Supplementary Fig. S1** Forest plot for subgroup analysis of overall survival in early-onset rectal cancer. Large tumor: >50 mm; Small tumor: ≤ 50 mm. HR, hazard ratio; CI, confidence interval; MA, mucinous adenocarcinoma; SRCC, signet ring cell carcinoma.


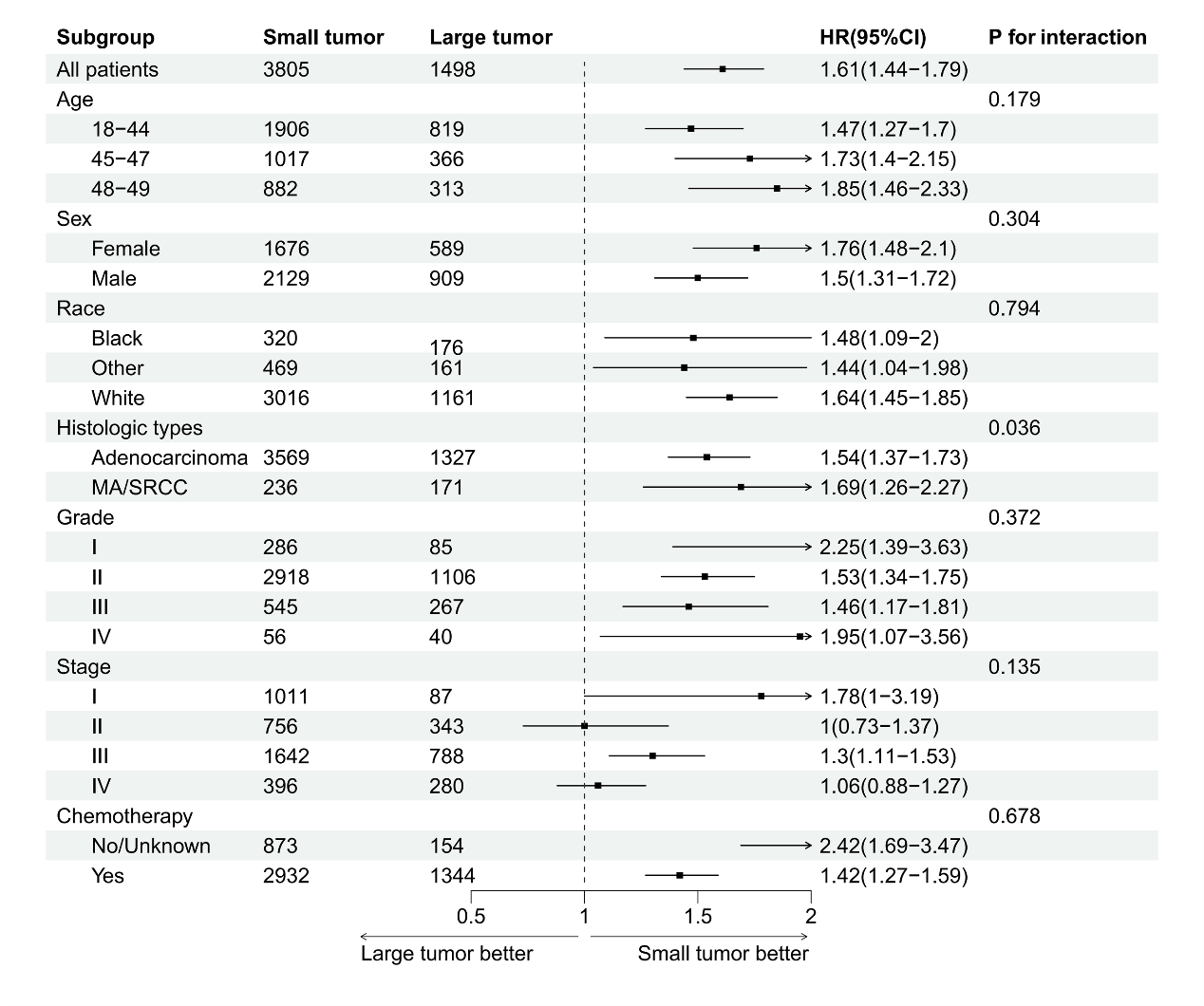


**Supplementary Fig. S2** Forest plot for subgroup analysis of cancer-specific survival in early-onset rectal cancer. Large tumor: >50 mm; Small tumor: ≤ 50 mm. HR, hazard ratio; CI, confidence interval; MA, mucinous adenocarcinoma; SRCC, signet ring cell carcinoma.

**Supplementary Table S1** Demographic and clinicopathological characteristics of patients with early-onset colon cancer for overall survival before and after PSM.

|  | **Before PSM** | | |  | **After PSM** | | |
| --- | --- | --- | --- | --- | --- | --- | --- |
| **Characteristics** | **Small tumor** | **Large tumor** | **SMD** |  | **Small tumor** | **Large tumor** | **SMD** |
| Overall | 10170 | 7381 |  |  | 6360 | 6360 |  |
| Age |  |  | 0.085 |  |  |  | 0.044 |
| 18-44 | 5153 (50.7) | 4033 (54.6) |  |  | 3411 (53.6) | 3382 (53.2) |  |
| 45-47 | 2685 (26.4) | 1865 (25.3) |  |  | 1677 (26.4) | 1600 (25.2) |  |
| 48-49 | 2332 (22.9) | 1483 (20.1) |  |  | 1272 (20.0) | 1378 (21.7) |  |
| Sex |  |  | 0.191 |  |  |  | 0.054 |
| Female | 5358 (52.7) | 3188 (43.2) |  |  | 2782 (43.7) | 2952 (46.4) |  |
| Male | 4812 (47.3) | 4193 (56.8) |  |  | 3578 (56.3) | 3408 (53.6) |  |
| Race |  |  | 0.066 |  |  |  | 0.069 |
| Black | 1509 (14.8) | 1269 (17.2) |  |  | 904 (14.2) | 1034 (16.3) |  |
| Other | 1168 (11.5) | 791 (10.7) |  |  | 797 (12.5) | 700 (11.0) |  |
| White | 7493 (73.7) | 5321 (72.1) |  |  | 4659 (73.3) | 4626 (72.7) |  |
| Histologic_types |  |  | 0.305 |  |  |  | 0.005 |
| Adenocarcinoma | 9475 (93.2) | 6161 (83.5) |  |  | 5715 (89.9) | 5706 (89.7) |  |
| MA/SRCC | 695 (6.8) | 1220 (16.5) |  |  | 645 (10.1) | 654 (10.3) |  |
| Grade |  |  | 0.226 |  |  |  | 0.079 |
| I | 750 (7.4) | 405 (5.5) |  |  | 449 (7.1) | 332 (5.2) |  |
| II | 7562 (74.4) | 4945 (67.0) |  |  | 4438 (69.8) | 4482 (70.5) |  |
| III | 1601 (15.7) | 1746 (23.7) |  |  | 1286 (20.2) | 1344 (21.1) |  |
| IV | 257 (2.5) | 285 (3.9) |  |  | 187 (2.9) | 202 (3.2) |  |
| Stage |  |  | 0.528 |  |  |  | 0.088 |
| I | 1912 (18.8) | 303 (4.1) |  |  | 299 (4.7) | 298 (4.7) |  |
| II | 2144 (21.1) | 2496 (33.8) |  |  | 2030 (31.9) | 1925 (30.3) |  |
| III | 4020 (39.5) | 2695 (36.5) |  |  | 2614 (41.1) | 2483 (39.0) |  |
| IV | 2094 (20.6) | 1887 (25.6) |  |  | 1417 (22.3) | 1654 (26.0) |  |
| Chemotherapy |  |  | 0.113 |  |  |  | 0.032 |
| No/Unknown | 3974 (39.1) | 2484 (33.7) |  |  | 2116 (33.3) | 2021 (31.8) |  |
| Yes | 6196 (60.9) | 4897 (66.3) |  |  | 4244 (66.7) | 4339 (68.2) |  |

Small tumor: ≤ 50 mm; Large tumor: > 50 mm. PSM, propensity score matching; SMD, standardised mean difference; MA, mucinous adenocarcinoma; SRCC, signet ring cell carcinoma.

**Supplementary Table S2** Demographic and clinicopathological characteristics of patients with early-onset colon cancer for cancer-specific survival before and after PSM.

|  | **Before PSM** | | |  | **After PSM** | | |
| --- | --- | --- | --- | --- | --- | --- | --- |
| **Characteristics** | **Small tumor** | **Large tumor** | **SMD** |  | **Small tumor** | **Large tumor** | **SMD** |
| Overall | 10121 | 7329 |  |  | 6523 | 6523 |  |
| Age |  |  | 0.085 |  |  |  | 0.082 |
| 18-44 | 5129 (50.7) | 4008 (54.7) |  |  | 3285 (50.4) | 3468 (53.2) |  |
| 45-47 | 2673 (26.4) | 1847 (25.2) |  |  | 1629 (25.0) | 1669 (25.6) |  |
| 48-49 | 2319 (22.9) | 1474 (20.1) |  |  | 1609 (24.7) | 1386 (21.2) |  |
| Sex |  |  | 0.191 |  |  |  | 0.051 |
| Female | 5331 (52.7) | 3163 (43.2) |  |  | 3153 (48.3) | 2988 (45.8) |  |
| Male | 4790 (47.3) | 4166 (56.8) |  |  | 3370 (51.7) | 3535 (54.2) |  |
| Race |  |  | 0.065 |  |  |  | 0.091 |
| Black | 1504 (14.9) | 1258 (17.2) |  |  | 879 (13.5) | 1061 (16.3) |  |
| Other | 1159 (11.5) | 780 (10.6) |  |  | 664 (10.2) | 731 (11.2) |  |
| White | 7458 (73.7) | 5291 (72.2) |  |  | 4980 (76.3) | 4731 (72.5) |  |
| Histologic_types |  |  | 0.305 |  |  |  | 0.017 |
| Adenocarcinoma | 9435 (93.2) | 6124 (83.6) |  |  | 5893 (90.3) | 5859 (89.8) |  |
| MA/SRCC | 686 (6.8) | 1205 (16.4) |  |  | 630 (9.7) | 664 (10.2) |  |
| Grade |  |  | 0.226 |  |  |  | 0.018 |
| I | 747 (7.4) | 403 (5.5) |  |  | 359 (5.5) | 361 (5.5) |  |
| II | 7532 (74.4) | 4916 (67.1) |  |  | 4580 (70.2) | 4536 (69.5) |  |
| III | 1586 (15.7) | 1728 (23.6) |  |  | 1385 (21.2) | 1412 (21.6) |  |
| IV | 256 (2.5) | 282 (3.8) |  |  | 199 (3.1) | 214 (3.3) |  |
| Stage |  |  | 0.529 |  |  |  | 0.006 |
| I | 1911 (18.9) | 303 (4.1) |  |  | 299 (4.6) | 303 (4.6) |  |
| II | 2134 (21.1) | 2490 (34.0) |  |  | 2031 (31.1) | 2040 (31.3) |  |
| III | 4000 (39.5) | 2678 (36.5) |  |  | 2501 (38.3) | 2504 (38.4) |  |
| IV | 2076 (20.5) | 1858 (25.4) |  |  | 1692 (25.9) | 1676 (25.7) |  |
| Chemotherapy |  |  | 0.113 |  |  |  | 0.018 |
| No/Unknown | 3958 (39.1) | 2469 (33.7) |  |  | 2036 (31.2) | 2092 (32.1) |  |
| Yes | 6163 (60.9) | 4860 (66.3) |  |  | 4487 (68.8) | 4431 (67.9) |  |

Small tumor: ≤ 50 mm; Large tumor: > 50 mm. PSM, propensity score matching; SMD, standardised mean difference; MA, mucinous adenocarcinoma; SRCC, signet ring cell carcinoma.

**Supplementary Table S3** Demographic and clinicopathological characteristics of patients with early-onset rectal cancer for overall survival before and after PSM.

|  | **Before PSM** | | |  | **After PSM** | | |
| --- | --- | --- | --- | --- | --- | --- | --- |
| **Characteristics** | **Small tumor** | **Large tumor** | **SMD** |  | **Small tumor** | **Large tumor** | **SMD** |
| Overall | 3815 | 1508 |  |  | 1426 | 1426 |  |
| Age |  |  | 0.090 |  |  |  | 0.040 |
| 18-44 | 1913 (50.1) | 824 (54.6) |  |  | 748 (52.5) | 775 (54.3) |  |
| 45-47 | 1018 (26.7) | 369 (24.5) |  |  | 355 (24.9) | 347 (24.3) |  |
| 48-49 | 884 (23.2) | 315 (20.9) |  |  | 323 (22.7) | 304 (21.3) |  |
| Sex |  |  | 0.096 |  |  |  | 0.026 |
| Female | 1681 (44.1) | 593 (39.3) |  |  | 551 (38.6) | 569 (39.9) |  |
| Male | 2134 (55.9) | 915 (60.7) |  |  | 875 (61.4) | 857 (60.1) |  |
| Race |  |  | 0.117 |  |  |  | 0.010 |
| Black | 320 (8.4) | 177 (11.7) |  |  | 149 (10.4) | 152 (10.7) |  |
| Other | 471 (12.3) | 163 (10.8) |  |  | 150 (10.5) | 153 (10.7) |  |
| White | 3024 (79.3) | 1168 (77.5) |  |  | 1127 (79.0) | 1121 (78.6) |  |
| Histologic_types |  |  | 0.183 |  |  |  | 0.007 |
| Adenocarcinoma | 3577 (93.8) | 1336 (88.6) |  |  | 1291 (90.5) | 1294 (90.7) |  |
| MA/SRCC | 238 (6.2) | 172 (11.4) |  |  | 135 (9.5) | 132 (9.3) |  |
| Grade |  |  | 0.142 |  |  |  | 0.056 |
| I | 286 (7.5) | 86 (5.7) |  |  | 77 (5.4) | 78 (5.5) |  |
| II | 2924 (76.6) | 1113 (73.8) |  |  | 1039 (72.9) | 1070 (75.0) |  |
| III | 549 (14.4) | 269 (17.8) |  |  | 280 (19.6) | 250 (17.5) |  |
| IV | 56 (1.5) | 40 (2.7) |  |  | 30 (2.1) | 28 (2.0) |  |
| Stage |  |  | 0.528 |  |  |  | 0.079 |
| I | 1012 (26.5) | 88 (5.8) |  |  | 86 (6.0) | 86 (6.0) |  |
| II | 757 (19.8) | 345 (22.9) |  |  | 374 (26.2) | 326 (22.9) |  |
| III | 1648 (43.2) | 793 (52.6) |  |  | 737 (51.7) | 776 (54.4) |  |
| IV | 398 (10.4) | 282 (18.7) |  |  | 229 (16.1) | 238 (16.7) |  |
| Chemotherapy |  |  | 0.340 |  |  |  | 0.025 |
| No/Unknown | 874 (22.9) | 157 (10.4) |  |  | 122 (8.6) | 132 (9.3) |  |
| Yes | 2941 (77.1) | 1351 (89.6) |  |  | 1304 (91.4) | 1294 (90.7) |  |

Small tumor: ≤ 50 mm; Large tumor: > 50 mm. PSM, propensity score matching; SMD, standardised mean difference; MA, mucinous adenocarcinoma; SRCC, signet ring cell carcinoma.

**Supplementary Table S4** Demographic and clinicopathological characteristics of patients with early-onset rectal cancer for cancer-specific survival before and after PSM.

|  | **Before PSM** | | |  | **After PSM** | | |
| --- | --- | --- | --- | --- | --- | --- | --- |
| **Characteristics** | **Small tumor** | **Large tumor** | **SMD** |  | **Small tumor** | **Large tumor** | **SMD** |
| Overall | 3805 | 1498 |  |  | 1417 | 1417 |  |
| Age |  |  | 0.092 |  |  |  | 0.082 |
| 18-44 | 1906 (50.1) | 819 (54.7) |  |  | 734 (51.8) | 778 (54.9) |  |
| 45-47 | 1017 (26.7) | 366 (24.4) |  |  | 393 (27.7) | 343 (24.2) |  |
| 48-49 | 882 (23.2) | 313 (20.9) |  |  | 290 (20.5) | 296 (20.9) |  |
| Sex |  |  | 0.096 |  |  |  | 0.008 |
| Female | 1676 (44.0) | 589 (39.3) |  |  | 504 (35.6) | 559 (39.4) |  |
| Male | 2129 (56.0) | 909 (60.7) |  |  | 913 (64.4) | 858 (60.6) |  |
| Race |  |  | 0.117 |  |  |  | 0.050 |
| Black | 320 (8.4) | 176 (11.7) |  |  | 148 (10.4) | 155 (10.9) |  |
| Other | 469 (12.3) | 161 (10.7) |  |  | 169 (11.9) | 147 (10.4) |  |
| White | 3016 (79.3) | 1161 (77.5) |  |  | 1100 (77.6) | 1115 (78.7) |  |
| Histologic_types |  |  | 0.185 |  |  |  | 0.022 |
| Adenocarcinoma | 3569 (93.8) | 1327 (88.6) |  |  | 1276 (90.0) | 1285 (90.7) |  |
| MA/SRCC | 236 (6.2) | 171 (11.4) |  |  | 141 (10.0) | 132 (9.3) |  |
| Grade |  |  | 0.145 |  |  |  | 0.056 |
| I | 286 (7.5) | 85 (5.7) |  |  | 96 (6.8) | 77 (5.4) |  |
| II | 2918 (76.7) | 1106 (73.8) |  |  | 1057 (74.6) | 1074 (75.8) |  |
| III | 545 (14.3) | 267 (17.8) |  |  | 235 (16.6) | 238 (16.8) |  |
| IV | 56 (1.5) | 40 (2.7) |  |  | 29 (2.0) | 28 (2.0) |  |
| Stage |  |  | 0.606 |  |  |  | 0.092 |
| I | 1011 (26.6) | 87 (5.8) |  |  | 85 (6.0) | 84 (5.9) |  |
| II | 756 (19.9) | 343 (22.9) |  |  | 370 (26.1) | 320 (22.6) |  |
| III | 1642 (43.2) | 788 (52.6) |  |  | 711 (50.2) | 769 (54.3) |  |
| IV | 396 (10.4) | 280 (18.7) |  |  | 251 (17.7) | 244 (17.2) |  |
| Chemotherapy |  |  | 0.345 |  |  |  | 0.022 |
| No/Unknown | 873 (22.9) | 154 (10.3) |  |  | 127 (9.0) | 136 (9.6) |  |
| Yes | 2932 (77.1) | 1344 (89.7) |  |  | 1290 (91.0) | 1281 (90.4) |  |

Small tumor: ≤ 50 mm; Large tumor: > 50 mm. PSM, propensity score matching; SMD, standardised mean difference; MA, mucinous adenocarcinoma; SRCC, signet ring cell carcinoma.
